# Managing the Health Effects of Temperature in Response to Climate Change: Challenges Ahead

**DOI:** 10.1289/ehp.1206025

**Published:** 2013-02-12

**Authors:** Cunrui Huang, Adrian G. Barnett, Zhiwei Xu, Cordia Chu, Xiaoming Wang, Lyle R. Turner, Shilu Tong

**Affiliations:** 1 School of Public Health, and; 2 Institute of Health and Biomedical Innovation, Queensland University of Technology, Brisbane, Queensland, Australia;; 3 Centre for Environment and Population Health, School of Environment, and; 4 Griffith Climate Change Response Program, Griffith University, Brisbane, Queensland, Australia; 5 CSIRO Climate Adaptation Flagship and CSIRO Ecosystem Sciences, Commonwealth Scientific and Industrial Research Organisation, Melbourne, Queensland, Australia

**Keywords:** adaptation, climate change, economic analysis, heat event, public health

## Abstract

Background: Although many studies have shown that high temperatures are associated with an increased risk of mortality and morbidity, there has been little research on managing the process of planned adaptation to alleviate the health effects of heat events and climate change. In particular, economic evaluation of public health adaptation strategies has been largely absent from both the scientific literature and public policy discussion.

Objectives: We examined how public health organizations should implement adaptation strategies and, second, how to improve the evidence base required to make an economic case for policies that will protect the public’s health from heat events and climate change.

Discussion: Public health adaptation strategies to cope with heat events and climate change fall into two categories: reducing the heat exposure and managing the health risks. Strategies require a range of actions, including timely public health and medical advice, improvements to housing and urban planning, early warning systems, and assurance that health care and social systems are ready to act. Some of these actions are costly, and given scarce financial resources the implementation should be based on the cost-effectiveness analysis. Therefore, research is required not only on the temperature-related health costs, but also on the costs and benefits of adaptation options. The scientific community must ensure that the health co-benefits of climate change policies are recognized, understood, and quantified.

Conclusions: The integration of climate change adaptation into current public health practice is needed to ensure the adaptation strategies increase future resilience. The economic evaluation of temperature-related health costs and public health adaptation strategies are particularly important for policy decisions.

Many studies have found that extreme temperatures are associated with an increased risk of illness and death (Basu 2009; Kovats and Hajat 2008; O’Neill and Ebi 2009). The associations between daily temperature and mortality are generally U-shaped, with lower mortality rates in a “comfort zone” of temperatures, and mortality rates rising progressively as temperatures become hotter or colder (Analitis et al. 2008; McMichael et al. 2008). The comfort zone varies between cities and is generally higher in warmer climates, suggesting adaptation to local climatic conditions.

Heat-related mortality is a growing public health concern because of climate change, population aging, and increasing urbanization (Luber and McGeehin 2008). Climate change is projected to increase global mean surface temperatures by 2–4.5°C with 76% probability, and over 4.5°C with 14% probability, by 2100 (Rogelj et al. 2012) and will increase the frequency and intensity of heat waves. These changes are likely to have a greater impact on persons living in old or poorly constructed houses, which offer less protection from the outside heat (Chapman et al. 2009) and those living without air conditioners (Kovats and Hajat 2008). Population aging may further amplify vulnerability: the elderly and those with preexisting disease are often more susceptible to heat (Basu 2009). A larger number of people living in cities may also contribute to additional heat-related health problems because of urban heat islands, an interaction between air pollution and heat, and higher concentrations of heat-susceptible people (Harlan and Ruddell 2011).

Managing the health effects of temperature in response to climate change is a global public health challenge (Hajat et al. 2010). Until now, most studies have focused on quantifying temperature–health relationships, characterizing vulnerable subgroups, identifying effect modifiers (e.g., air pollution, socioeconomic status), and projecting future health impacts under climate change scenarios (Basu 2009; Hajat and Kosatsky 2010; Huang et al. 2011a; O’Neill and Ebi 2009; Vandentorren et al. 2006). There has been less research on minimizing the health risks of temperature exposure, and long-term strategies to address the health effects of temperature have not been sufficiently considered in public health practice and activities (Ebi 2011). Here we discuss how public health organizations should implement adaptation strategies and how to improve the evidence base required to make the economic case for policies that will protect health from heat events and climate change.

## Public Health Adaptation to Heat Events in Response to Climate Change

Public health adaptation refers to any short- or long-term strategies that can reduce adverse health effects or enhance resilience in response to climate change and associated weather extremes as well as exploit any beneficial opportunities (Frumkin et al. 2008; Hess et al. 2012; Huang et al. 2011b). Resilience is the ability of a system to timely and effectively prepare for, respond to, and recover from the consequences of disasters (Ebi 2011). Adaptation strategies to deal with heat events and climate change fall into two categories: reducing the heat exposure and managing associated health risks.

*Reducing heat exposure.* During extreme heat events there is limited potential for people to improve their physiological adaptive capacity [Hajat et al. 2010; World Health Organization (WHO) 2009]. Studies have found that heat waves at the start of summer have a greater impact on health than heat waves later in summer, which may be due to inadequate acclimatization early on (Anderson and Bell 2011). It is therefore important to plan ahead ways to decrease exposure to heat, particularly for susceptible persons.

Access to cooling. Reducing vulnerable groups’ heat exposure is an important protective measure during a heat wave. Spending even a few hours in a cool environment has been shown to be strongly protective for reducing heat-related illnesses and deaths (Luber and McGeehin 2008). Epidemiological studies of heat-related mortality have shown a strong protective effect due to air conditioning (O’Neill et al. 2009).

Some at-risk persons, particularly the elderly, may not have or use air conditioning during heat waves (PricewaterhouseCoopers 2011; Sheridan 2007). This may be because of a failure to recognize the need for air conditioning or their reluctance to use it given the high electricity costs. Therefore, it would be of value to educate vulnerable groups about how best to use air conditioning and to provide financial assistance to low-income groups.

Some researchers caution against the widespread use of air conditioning (Maller and Strengers 2011). Air conditioners emit heat during use, increase outdoor temperature, and often use electricity generated by fossil fuel–burning power plants (O’Neill et al. 2009). Demands for electricity during heat waves can burden power infrastructure, and even lead to large-scale power failures. Recent evidence from the United States showed how heat-related deaths can dramatically increase during power failures (Anderson and Bell 2012).

Increasing the use of air conditioners will also further increase greenhouse gas (GHG) emissions and consequently contribute to accelerated climate change and deteriorating local air quality. Government-run cooling centers may reduce risk for the potentially large number of people unable to afford air conditioning in their own homes. However, the effectiveness of cooling centers relies on people having transportation and recognizing when they should go to the centers (O’Neill et al. 2009).

Building design. People spend about 90% of their lives indoors and tend to stay indoors during hot weather, so indoor temperature is of particular importance for preventing heat-related health effects (Chapman et al. 2009). Indoor temperature is a function of outdoor temperature, solar radiation, building insulation and ventilation, and the building’s ability to dissipate stored heat (Institute of Medicine 2011). Adaptation to heat events can be improved by adjusting indoor temperature through improved building design so that the indoor temperature is more often in a comfortable range regardless of the outdoor temperature.

Building orientation, design, and materials can all influence the impact of outdoor heat on indoor temperatures. Insulation can act as a barrier to hot air and is essential to keep homes warm in winter and cool in summer. As a long-term goal, public health measures should advocate for improving thermal insulation in old and poorly constructed buildings (Vandentorren et al. 2006). These measures may be costly and time-consuming, but their implementation is necessary given the current considerable excess deaths due to cold temperatures and the expected increasing frequency of heat waves (Analitis et al. 2008; Anderson and Bell 2011).

Urban planning. Cities are usually warmer than the surrounding countryside, a phenomenon known as the urban heat island effect (Smargiassi et al. 2009). Densely packed buildings absorb solar radiation during the day and then block the surface heat from radiating at night (Luber and McGeehin 2008). Also, many cities have decentralized in recent decades, creating urban sprawl with low-density land use and heavy reliance on cars. A U.S. study found that these sprawling cities had double the rate of increase in the annual number of extreme heat events compared with more compact cities (Stone et al. 2010).

Urban heat islands and urban sprawl may be an inevitable consequence of development, but appropriate planning can play an important role in reducing vulnerability, building resilience, and promoting health (Stone et al. 2010). Reductions in heat-related deaths can be achieved in a number of ways. High-density communities can be developed with mixed land use and good connectivity to promote active transport (walking and cycling assist in reducing cardiovascular disease, conditions that place individuals at risk in heat events). The amount of green spaces and trees can be increased in cities to provide shade and improve air quality. Trees and vegetation also help reduce daytime temperatures and the urban heat island effect through the process of evapotranspiration. In terms of building design, the use of reflective paving and roofing materials can be promoted, as well as increasing the amount of ventilation and air flow between buildings. Also, providing adequate public spaces through urban planning can promote social cohesion, which can reduce health risks faced by people who are otherwise socially isolated (Luber and McGeehin 2008; WHO 2009).

*Managing the health risks*. Public health efforts to deal with heat-related health problems require a variety of actions, including real-time data surveillance and early warnings, health care system preparedness, and public health awareness campaigns.

Data surveillance and early warning. There is a need to develop timely and effective surveillance systems to facilitate public health responses to heat events (Leonardi et al. 2006). Real-time data surveillance can provide early detection of heat-related health effects by informing decision makers about abnormal numbers of adverse health outcomes. During hot weather, the most useful real-time health data are deaths, emergency department visits, and ambulance calls (WHO 2009). Health data for surveillance need to be available promptly because increases in mortality and morbidity from heat events occur quickly after exposure (Leonardi et al. 2006). Ambulance calls may have the most immediacy, and they are sensitive to hot weather (Turner et al. 2012).

In response to heat waves, heat–health warning systems are becoming more widespread (O’Neill and Ebi 2009; WHO 2009). The essential elements of a heat–health warning system are identifying locally relevant extreme weather, designing sensitive and specific trigger alerts, monitoring weather forecasts, and putting appropriate response actions in place (Ebi and Schmier 2005). However, there has been little research to evaluate the effectiveness of these systems. Although they aim to reduce heat-related illness and death, it is difficult to attribute mortality or morbidity reductions directly to a new warning system because there will be other changes over time, such as increased air conditioning use, improved public awareness, and better health care system preparedness.

Health care system preparedness. No matter how good a heat–health warning system is at identifying dangerous heat waves, it will not save lives if there are no effective public health interventions that are triggered by the warning. The delivery of health care services can be challenging during extreme heat events. Health care providers need to maintain a high level of services to help those affected by the heat and support the community in general. However, working in high-temperature environments can potentially affect the health and safety of workforces. When a severe heat wave occurs, health care workers may be reluctant to work when the situation poses a possible threat to their own health and safety. Such reluctance further stresses an already overcrowded and stretched health care system (Smith et al. 2008). Hence, hospitals, clinics, and nursing homes may need to add staff and increase their rotation during hot weather.

Social factors such as social isolation can also influence vulnerability to heat (Hajat et al. 2010; Kovats and Hajat 2008). Health care delivery should be suited to the needs of the most vulnerable groups through coordination between health departments, social services, and other community organizations. The best plan is to identify the most feasible and appropriate options based on the structure of local health care and social service systems (WHO 2009).

Awareness and education. Responding effectively to heat events requires more than institutional arrangements. It requires individuals to understand what heat events are, what health effects they can have, and what they themselves can do to minimize these effects (PricewaterhouseCoopers 2011). Public health campaigns need to not only create greater awareness of the dangers of heat to inform individuals, but also provide practical tips on how to reduce their risks (Sheridan 2007). These tips might include keeping hydrated, avoiding alcohol, wearing light clothing, taking cold showers, checking on older friends and neighbors, and recognizing the symptoms of heat stress.

Key messages should be targeted at the public, but also adjusted to the needs of specific vulnerable groups such as the elderly or high-risk occupational groups. Information for these vulnerable groups should contain practical tips and important contact details for social and emergency services (Hajat et al. 2010; WHO 2009). It is also important to design training programs for doctors, nurses, and other health care professionals to enable them to identify heat-related health problems and be familiar with the most appropriate treatments (WHO 2009).

## Challenges and Opportunities

Research on the health effects of temperature has increased rapidly in recent years, but investment in specific health protection measures (e.g., cooling centers, home insulation) has failed to keep pace. Policy decisions that are designed to reduce the health risks of extreme temperatures and climate change involve making trade-offs concerning the use of scarce financial resources (Hutton 2011). Economic efficiency is an important criterion for decision making. However, economic analysis of temperature-associated health costs and public health adaptation options have been largely absent from both the scientific literature and public policy discussions (Knowlton et al. 2011).

*Temperature-associated health costs*. It is useful to estimate the health costs of temperature exposure and climate change in monetary terms. Estimates of the economic costs can be used in cost–benefit analyses for comparing proposed adaptation strategies (Hutton 2011; Markandya and Chiabai 2009). However, the valuation of a life is a controversial issue and one with irrationalities. For example, society is usually willing to save identified lives such as sailors stranded in mid-ocean or children in need of expensive surgery, but it is often reluctant to invest in preventive measures that would benefit more people. An example is when the lives saved are anonymous, as would be the case when funding a home insulation program. Insulation prevents deaths and hospitalizations from either hot- or cold-related stress (Chapman et al. 2009), but these invisible savings are of little value to politicians trying to impress the voting public.

To distinguish the value of an anonymous life from the life of an identified person, the concept of “statistical life” has been developed for the purposes of policy making. For estimating the value of a statistical life, there has been growing interest in the development of health outcome measures, such as years of life lost (YLL), disability-adjusted life year (DALY), and quality-adjusted life year (QALY) (Lopez et al. 2006). A variety of methods have been used to value a statistical life, such as human capital and willingness-to-pay approaches. In Australia, a threshold of AUD $40,000 per year of life is often used by Australian resource allocation committees, for example, the Medical Services Advisory Committee and the Pharmaceutical Benefits Advisory Committee (George et al. 2001). It means that interventions that save 1 year of life for every AUD $40,000 invested are cost effective and should be funded.

Case study in Brisbane, Australia. The health costs of temperature exposure and climate change have never been fully calculated in Australia. Furthermore, there are no widely accepted estimates available on the costs associated with these effects. According to our recent study in Brisbane (Huang et al. 2012), around 6,500 YLL were lost per year due to current temperature-related deaths based on data for 1996–2003. There were 1,500 YLL due to hot temperatures and 5,000 YLL due to cold temperatures. For a future 2°C temperature increase in 2050, we projected an increase of around 400 temperature-related YLL, and for a 4°C increase we projected an increase of around 3,000 YLL, relative to the baseline period 1996–2003.

Combining the estimated years of life lost with the economic threshold of AUD $40,000 per year of life shows that the future health costs of ambient temperature and climate change could be enormous in Brisbane (AUD $278 million for a 2°C temperature increase and AUD $393 million for a 4°C temperature increase) (Table 1). This means that policy makers should be willing to invest in adaptation strategies that would reduce temperature-related deaths. Money spent now would also be of immediate benefit because the current costs of temperature-related deaths are AUD $263 million per year in Brisbane (Table 1), and we would expect similar costs in other large cities. This immediate benefit may be particularly important for politicians who favor policy decisions that will return benefits before their next reelection campaign.

**Table 1:** Monetary estimates (95% CI) of the annual health costs due to climate change’s effects on ambient temperatue in Brisbane, Australia (AUD $ million

	Hot days	Cold days	Whole year
Baseline	61 (49, 73)	202 (150, 253)	263 (212, 312)
1°C increase
Projection	101 (83, 119)	158 (114, 200)	259 (213, 304)
Change	41 (34, 47)	–44 (–53, –35)	–4 (–15, 8)
2°C increase
Projection	159 (131, 184)	119 (83, 154)	278 (232, 322)
Change	98 (82, 114)	–83 (–99, –65)	15 (–6, 39)
3°C increase
Projection	235 (196, 273)	87 (58, 115)	322 (272, 374)
Change	174 (146, 202)	–115 (–138, –90)	59 (25, 97)
4°C increase
Projection	332 (278, 385)	61 (38, 83)	393 (332, 456)
Change	271 (227, 316)	–142 (–171, –110)	130 (80, 184)
Baseline for the years 1996–2003 centered on 2000; projection for the years 2046–2053 centered on 2050. We assumed that climate change will cause increasing average temperatures but no change in variability. We simulated future daily temperatures by adding 1–4°C to the observed daily temperature data. The total population for Brisbane was 896,649 in 2001. Hot days are a daily mean temperature > 23°C, and cold days < 23°C. We chose this temperature because it was the turning point in the association between temperature and years of life lost for both men and women (Huang et al. 2012). The dollar values are based on AUD $40,000 per year of life. Average exchange rate in August 2012: 1 AUD = 1.03 USD.			

*Health economic evaluation*. Health costs alone are of limited use for decision making. Decisions to implement adaptation strategies should rest on a sound economic evaluation. How much adaptation might cost and the magnitude of related benefits are issues that are particularly relevant to policy decisions made under budgetary allocations (Agrawala and Fankhauser 2008). Therefore, research is required not only on the health costs of temperature, but also on the costs and benefits of the associated adaptation options.

The scientific literature on the costs of adaptation to protect health from heat events and climate change remains sparse. Estimates of savings to be made from sound investments in preventing temperature-related health effects have not been adequately conducted. In the United States, Ebi et al. (2004) reported that the cost of running a heat–health warning system for Philadelphia was relatively small (USD $210,000) compared with the benefits of saving 117 lives (USD $468 million) over the 3-year period of 1995–1998. Such economic evidence can help inform policy makers as to where the greatest health gains can be achieved through sound investments in adaptation measures.

Estimating climate change adaptation costs can be difficult. Many adaptation actions are embedded within responses to a broader set of social and environmental incentives (e.g., increasing green space to promote physical activity), making it difficult to isolate the adaptation costs. In addition, adaptation costs may increase greatly if actions to improve adaptive capacity are also included (e.g., investments in education, nutrition, and health care system infrastructure).

There are no accepted methods for assessing the benefits of adaptation strategies. Uncertainty about the potential health effects of climate change implies that most of the adaptation benefits should be interpreted as expected, not guaranteed, benefits. Another issue is that the long-term nature of climate change makes timing an important part of adaptation decisions (Agrawala and Fankhauser 2008). Timely or early adaptation may not occur if there is no perception of immediate benefit. Policy makers must compare costs and benefits that are experienced at different times because often the costs of adaptation will be incurred in the near future although the benefits will be spread over a much longer time (e.g., planting trees in cities although the benefits of increased shade are realized years later) (Markandya and Chiabai 2009).

*Health co-benefits of climate change policies*. The development of climate change mitigation and adaptation policies are an important step toward building resilience to heat events and climate change, but in many jurisdictions challenges and trade-offs are already apparent in the implementation stage. Implementing these policies requires an initial financial investment, which local governments and decision makers may be unwilling to make, particularly in a depressed economy (Harlan and Ruddell 2011). Perhaps the biggest challenge to implementing these policies is to balance the costs and benefits of a given strategy that draws on limited economic resources.

Many climate change policies can improve public health via co-benefits, and research is needed on how much these health co-benefits decrease the overall costs of such policies. For example, some strategies both reduce GHG emissions and cool cities through changes in the built environment (e.g., urban green space planning, energy-efficient building materials, active transportation) (Younger et al. 2008). Implementing these strategies can bring about substantial health co-benefits, including reductions in heat-related illnesses and lifestyle-related diseases (e.g., reducing GHG emissions by encouraging active transport, which then reduces the risk of cardiovascular disease) (Haines et al. 2009; Harlan and Ruddell 2011).

Because the health co-benefits of climate change policies are likely to decrease the costs to society, it is necessary to quantify the extent of the savings (Haines et al. 2009; Roberts 2009). However, although the health co-benefits of climate change policies are increasingly recognized by public health researchers, they are not widely appreciated by policy makers and the general public. Hence, the public health research community must ensure that the health co-benefits of climate change policies are recognized, understood, and quantified and that this information is distributed to relevant stakeholders (Roberts 2009).

## Conclusions

Heat events are hazardous to health; a growing literature is reporting increases in mortality and morbidity during hot weather. Public health responses require a range of actions, including timely public health and medical advice, improvements to housing and urban planning, early warning systems, and the assurance that health care and social systems are ready to act. Explicit consideration of climate change in existing public health activities is needed to ensure they have maximum effectiveness for increasing future resilience. However, adaptation actions may have substantial financial implications for the health sector.

Policy decisions that are designed to reduce the health risks of extreme temperatures and climate change involve making trade-offs concerning the use of limited resources. Making better decisions will depend on an evidence-based economic assessment of temperature-related health costs and public health adaptation options.
